# Effect of Cyclic Stretch on Vascular Endothelial Cells and Abdominal Aortic Aneurysm (AAA): Role in the Inflammatory Response

**DOI:** 10.3390/ijms20020287

**Published:** 2019-01-12

**Authors:** Martina Ramella, Giulia Bertozzi, Luca Fusaro, Maria Talmon, Marcello Manfredi, Marta Calvo Catoria, Francesco Casella, Carla Maria Porta, Renzo Boldorini, Luigia Grazia Fresu, Emilio Marengo, Francesca Boccafoschi

**Affiliations:** 1 Department of Health Science, University of Piemonte Orientale (UPO), via Solaroli 17, 28100 Novara, Italy; martina.ramella@med.uniupo.it (M.R.); gbertozzi.gb@gmail.com (G.B.); luca.fusaro@med.uniupo.it (L.F.) maria.talmon@med.uniupo.it (M.T.); marta.catoira@uniupo.it (M.C.C.); renzo.boldorini@med.uniupo.it (R.B.); luigia.fresu@med.uniupo.it (L.G.F.); 2 TissueGraft s.r.l., Spin-off of University of Piemonte Orientale (UPO), via Canobio 4/6, 28100 Novara, Italy; 3 ISALIT s.r.l., Spin-off of DISIT, University of Piemonte Orientale (UPO), 15121 Alessandria, Italy; marcello.manfredi@uniupo.it; 4 Department of Sciences and Technological Innovation, University of Piemonte Orientale (UPO), 15121 Alessandria, Italy; emilio.marengo@uniupo.it; 5 Vascular Surgery Unit, Ospedale Maggiore della Carità, 28100 Novara, Italy; fcasella1973@gmail.com (F.C.); carla.porta@maggioreosp.novara.it (C.M.P.)

**Keywords:** cardiovascular diseases, abdominal aortic aneurysm, oxidative stress, inflammation, calcification, cyclic stretch

## Abstract

Abdominal aortic aneurysm (AAA) is a focal dilatation of the aorta, caused by both genetic and environmental factors. Although vascular endothelium plays a key role in AAA progression, the biological mechanisms underlying the mechanical stress involvement are only partially understood. In this study, we developed an *in vitro* model to characterize the role of mechanical stress as a potential trigger of endothelial deregulation in terms of inflammatory response bridging between endothelial cells (ECs), inflammatory cells, and matrix remodeling. In AAA patients, data revealed different degrees of calcification, inversely correlated with wall stretching and also with inflammation and extracellular matrix degradation. In order to study the role of mechanical stimulation, endothelial cell line (EA.hy926) has been cultured in healthy (10% strain) and pathological (5% strain) dynamic conditions using a bioreactor. In presence of tumor necrosis factor alpha (TNF-α), high levels of matrix metalloproteinase-9 (MMP-9) expression and inflammation are obtained, while mechanical stimulation significantly counteracts the TNF-α effects. Moreover, physiological deformation also plays a significant role in the control of the oxidative stress. Overall our findings indicate that, due to wall calcification, in AAA there is a significant change in terms of decreased wall stretching.

## 1. Introduction

Abdominal aortic aneurysm (AAA) is a degenerative disease caused by permanent dilatation of the aorta in the abdominal infrarenal tract [1]. AAA annual incidence is 0.4–0.67% in western population [2,3,4], while the prevalence is 4–8% [5,6,7], and it is more common in men than in women. Although the exact etiology of AAA is unknown, there are several risk factors related to AAA development such as male gender, age (≥ 50 years old), smoking habits, atherosclerosis, and hypertension, and some genetic factors [8,9], which also involve lipoproteins [10].

Aneurysm can develop slowly, even silently and asymptomatically until the rupture occurs, causing massive hemorrhage with an elevated risk of death due to hypovolemic and hemorrhagic shock [11]. Extracellular matrix (ECM) degradation and oxidative stress represent hallmarks of AAA progression [12]. Calcification is commonly found within the aneurysm wall and leading to wall stiffening, and eventually to its rupture [13]. Moreover, increased mechanical stresses due to turbulent flow within the wall contribute to AAA progression and rupture [14]. The ability of a vascular wall to relax and passively contract depending on pressure changes and blood flow is a physiological characteristic of large elastic arteries, and it is defined as ‘compliance’. Bloodstream in the vascular compartment follows the laws of laminar flow; laminar flow is altered by the reduction of flow velocity (blood stasis), as well as by the fluctuation of flow (turbulence). The classical turbulent flow causes endothelial damage as it leads to the generation of flows that are contrary to the direction of the circulatory current, also generating pockets of stasis [15]. In arterial aneurysms, especially those with saccular morphology, there may be a slowing down of flow until blood stasis. This stasis generates on the one hand the possible formation of parietal thrombosis and on the other the alteration of the endothelium with the subsequent deposition of calcifications in the medium tunic [16,17].

Blood flow in the arterial aneurysm follows La Place’s law, which explains how the parietal tension (T) depends on the transmural pressure (Ptm), wall thickness (d), and radius of the container (r) according to the equation

T=(Ptm∗r)/d.

If the volume increases, the parietal voltage increases. In fact, if the vessel expands, the increase in the radius coupled with the decrease in the wall thickness increases the tension required to counteract the transmural pressure. Blood vessels function as viscoelastic tubes, and they respond to a transmural pressure gradient as a function of blood vessel wall composition. As vessel wall is subjected to a transmural pressure gradient, a portion of the intraluminal energy is used to stretch the fibers within the wall. The energy stored within the blood vessel fibers is later released back into the system, upon closure of the aortic valve. As intraluminal pressure oscillates, the constant loading and unloading of the fibers in the vessel wall results in a change in diameter of the blood vessel, which is noted clinically as a palpable pulse. Although vascular endothelial cells (ECs) and vascular smooth muscle cells (vSMCs) are exposed to both types of mechanical forces, shear stress resulting from blood flow is sensed mainly by ECs [18], whereas both ECs and vSMCs are subjected to cyclic stretch resulting from pulsatile pressure. In pathological remodeling, ECs can be influenced on structural as well as functional aspects. Firstly, they can change morphology, acquiring a bigger size and an irregular shape; they can also lose most of their regulatory roles. Endothelial layer can become more permeable, allowing the transit of several substances and vSMC infiltration. Endothelium shows inflammatory features, and it is characterized by the hyperexpression of proinflammatory cytokines (interleukins as IL-1, IL-6, and tumor necrosis factor alpha, TNF-α), proinflammatory chemokines (IL-8, monocyte chemoattractant protein 1, MCP-1, and regulated upon activation, normal T-Cell expressed- and secreted, RANTES) [19,20] and cell adhesion molecules (CAMs) such as selectins and integrins, [21] with a significant production of reactive oxygen species (ROS), in particular hydrogen peroxide (H_2_O_2_), superoxide (O_2_^−^), and hydroxyl radical (.OH). [22,23] Deregulation of NADPH oxidase (NOX), xanthine oxidase (XO), superoxide dismutase (SOD), thioredoxin (TRX), and catalase results in extreme ROS production. [24,25] Moreover, ROS regulate ECM remodeling, acting directly on matrix metalloproteinases (MMPs) up-regulation, activating nuclear factor kB (NF-kB) and activator protein (AP-1). [26,27,28] The aim of this work is to clarify the role of vascular wall stretching in the maintenance of vascular physiology reproducing *in vitro* the pathological dilatation (static and 5%), due to calcification, and physiological (10%) cyclic (1 Hz) stretch of the vessel wall, in order to study the effects of mechanical stress on ECs functionality in terms of inflammation, matrix remodeling, and oxidative stress production.

## 2. Results

### 2.1. Relationship between Wall Stress and Degree of Calcification

**Figure 1:**
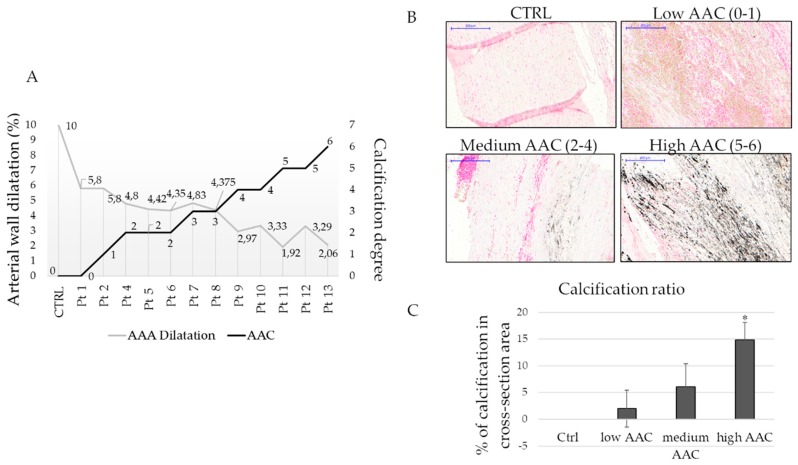
Wall stress and calcification correlation. (**A**) Graph showing the relation between abdominal aortic aneurysm (AAA) dilatation and calcification (AAC, aortic aneurysm calcification); (**B**) Representative images of calcification degree evaluated by Von Kossa staining in paraffin-embedded tissues, scale bar 200 µm; (**C**) Quantification of calcium content in healthy tissues (CTRL) and aneurysm tissues evaluated by ImageJ software. * indicates *p* ≤ 0.05 respect to control.

Patient-specific AAA geometries are reconstructed, and structural analysis is performed to calculate the wall stresses of the AAA models and their calcification. In Figure 1A is shown how the wall dilatation changes in relation to the amount of calcification. Retrospective analyses of literature show that the aortic dilatation in healthy donors is 10% considering the ratio between systole and diastole; this data is confirmed also by our measures on healthy controls. In fact, in presence of aneurysm the dilatation is less than 10%, and it decreases when the calcification index increases (Figure 1A). Control aortas and AAA sections are stained with Von Kossa to confirm the calcification degrees obtained by the measurements with computed axial tomography (CAT) analysis. As expected, controls do not show any calcium accumulation, while AAA tissues present dark spots (calcium deposition) in particular in the medial layer, in a directly proportional manner to the degree of calcification, as shown by related quantification (Figure 1B,C). 

### 2.2. Correlation between Calcium Deposition and Inflammation

**Figure 2:**
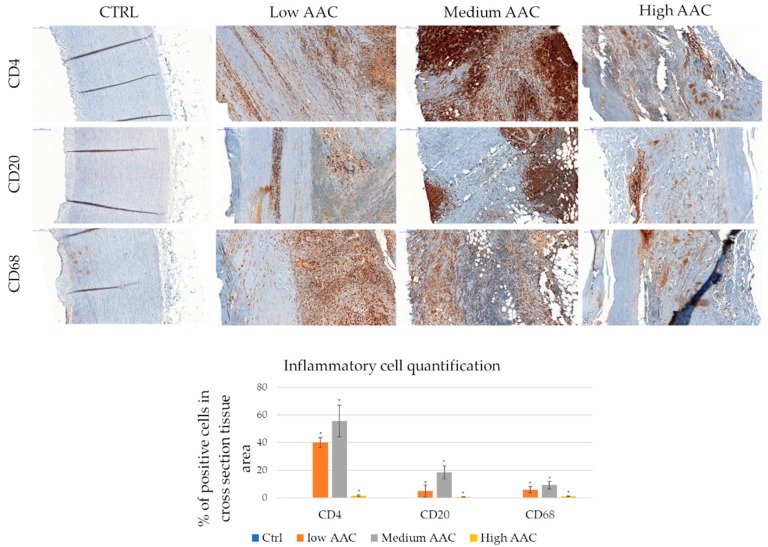
Inflammatory cell infiltration in AAA. Representative immunohistochemistry for anti-Cluster of differentiation 4 (CD4), CD8, CD68 staining. CD4+ is performed for T-helper lymphocytes, CD8+ for T-killer, and CD68+ for monocytes-macrophage. Healthy aorta, represented on the left, is negative for inflammatory cells infiltration. Scale bar 200 µm. Inflammatory cell quantification is represented as a percentage of positive cell covered area. * indicates *p* ≤ 0.05 respect to controls (healthy tissues).

Knowing that AAA is characterized by an inflammatory condition, different inflammatory markers related to inflammatory cells are investigated, in particular CD4 (Cluster of differentiation 4), CD20, and CD68, respectively for T-helper lymphocyte, B-cell, and macrophage identification. Control tissues are negative for all the markers, while in the low and medium aortic aneurysm calcification (AAC) index all these markers are significantly represented. High AAC index tissues show a decrease of the inflammatory population correlated to CD4, CD20, and CD68 markers with respect to low and medium indexes. (Figure 2). 

**Figure 3:**
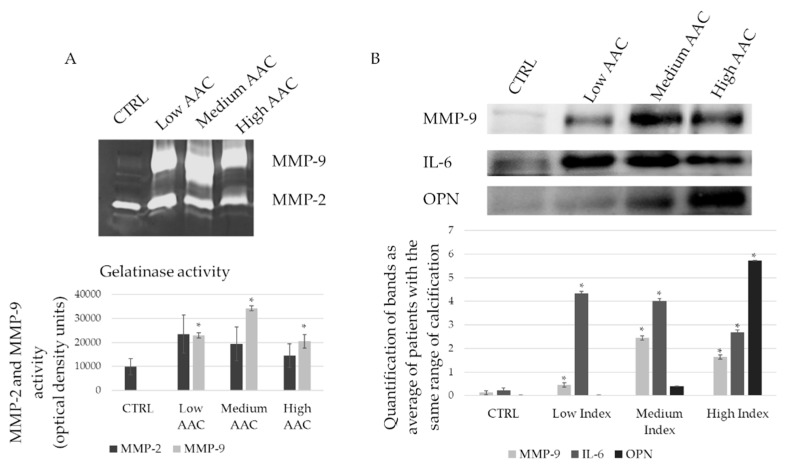
Matrix remodelling, inflammation and calcification in AAA tissues. (**A**) Gelatin zymography performed on tissue lysates of patients with different degree of calcification and the respective quantification of matrix metalloproteinase -2 and -9 (MMP-2 and MMP-9) activity; (**B**) Immunoblot on MMP-9, interleukin-6 (IL-6), and osteopontin (OPN) on tissue lysates of patients with different degree of calcification. The graph shows the relative quantification. * statistically significant with respect to control *p* < 0.05.

Other pro-inflammatory and calcification markers were investigated on tissue lysate, such as MMP-9, IL-6, and osteopontin (OPN) (Figure 3A,B). For all the considered markers, we obtain a significant difference between patients and controls. Differences related to the degree of calcification are appreciable: considering MMP-9, samples with medium index of calcification reach the higher expression as observed by western blot assay as well as the higher proteolytic activity, as indicated by zymography assay. The high calcification index indicates also the terminal phase of the degradation, thus MMP-9 results decreased in terms of protein expression and proteolytic activity. Considering IL-6 as an inflammatory marker, it decreases with the progression of calcium accumulation confirming the previous data, particularly, it has the same trend as MMP-9. As expected, patients with high calcification index have an increased expression of OPN, which has an osteogenic activity (Figure 3B). No significant differences are observed between healthy donors and patients in all AAC indices for MMP-2 activity (Figure 3A).

### 2.3. Oxidative Stress Proteins Are Overexpressed in AAA

The proteomic analysis of aortic abdominal aneurysms and control healthy vessel tissues was performed with liquid chromatography-mass spectrometry (LC-MS) in order to investigate the modulation of some proteins related to the oxidative stress processes. Table 1 reports the identities, the modulation and the biological functions of seven proteins resulted over expressed in the AAA respect with the healthy tissues. The enrichment of all these proteins indicates that the oxidative stress pathway is strongly involved in the AAA disease and that is particularly upregulated in the aortic abdominal vessel tissue. 

Catalase (CATA) and superoxide dismutase (Mn), mitochondrial (SODM) present a fold change of 2.51 and 6.95 respectively: these two proteins are involved in the cellular response to oxidative stress pathway, as a result of the exposure to high levels of reactive oxygen species. SODM is also involved in the removal of superoxide radicals and in oxidation-reduction processes. Regarding the last biological function, we found that protein disulfide-isomerase (PDIA1), isoform H14 of myeloperoxidase (PERM), ceruloplasmin (CERU), ferritin heavy chain (FRIH), and ferritin light chain (FRIL) are all strongly upregulated (fold change of 2.50, 3.04, 12.83, 21.83, and 26.43) in the AAA tissue.

**Figure 4:**
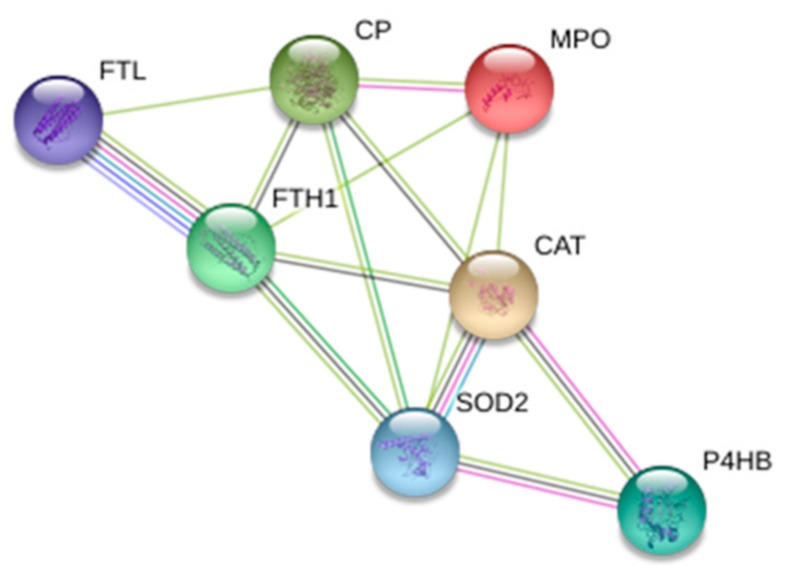
STRING network analysis of oxidative stress-related proteins that are over expressed in AAA vessel tissue respect with normal tissue. A proteome interactomic map was performed using the STRING tool for obtaining cross correlation information. Homo sapiens was selected as a reference organism. Different colored lines represent the existence of different types of evidence. A yellow line indicates text-mining evidence; a purple line, experimental evidence, a cyan line indicates association in curated database and black lines indicates co-expression data.

A protein–protein interaction analysis (Figure 4) of oxidative stress-related proteins was performed using STRING software. The network analysis showed a high-connected network among these proteins. Ferritin light chain and Ferritin heavy chain are co-expressed, showed evidence in experimental and association in curated database. Also superoxide dismutase, catalase, and protein disulfide-isomerase showed the same connections. The other linked proteins mainly showed text-mining evidence.

### 2.4. Mechanical Stimulation Drives ROS/Superoxide Production in EA.hy926

**Figure 5:**
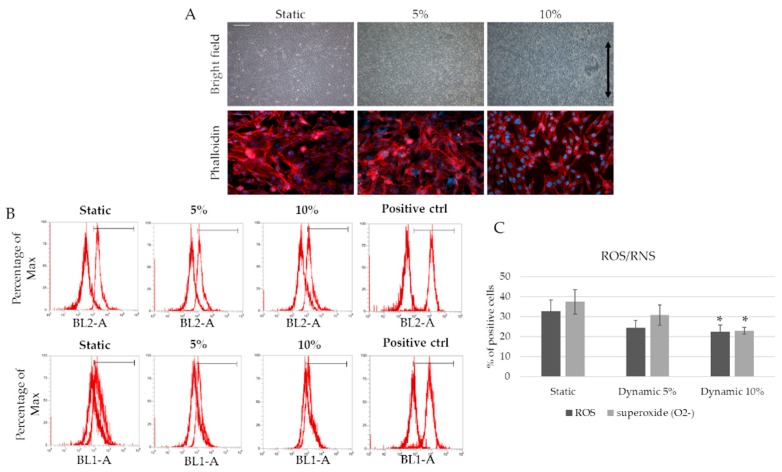
Mechanical strain drives reactive oxygen species (ROS) and reactive nitrogen species (RNS) production. (**A**) EA.hy926 after three days of 5% and 10% strain. Scale bar 200 µm. Phalloidin was used to observe cell morphology. Scale bar 25 µm; (**B**) Fluorescence-activated cell sorting (FACS) analyses for ROS and RNS production. Dark red represents unstained cells, while light red represents the experimental samples. BL2-A for superoxide detection; BL1-A for ROS detection; (**C**) graph of ROS/RNS production * statistically significant with respect to static samples. *p* < 0.05.

Since mechanical stimulation seems to play a key role in driving the inflammatory response and matrix remodeling, an in vitro dynamic model with physiological and pathological strain parameters, culturing an endothelial cell line (EA.hy926), was used. The effect of mechanical stimulation (5–10% deformation, 1 Hz frequency) is evaluated in the presence and the absence of an inflammatory stimulus (TNF-α 50 ng/mL) after three days of culture. Figure 5A shows cell morphology when 5% and 10% mechanical strain is applied for three days. While no differences are detected in terms of cell viability among the samples, 10% strain cultured cells display an elongated and oriented shape in the strain direction, whereas random orientation is observed in the other samples (static and 5% strain). In terms of ROS/RNS (reactive nitrogen species) production, the difference between 10% strained cells (physiological) and 5% strained or static cells (pathological) is confirmed, with lower ROS/RNS production in physiological conditions (Figure 5B,C). 

### 2.5. Strain Affects CD62E Expression and Monocytes Adhesion

**Figure 6:**
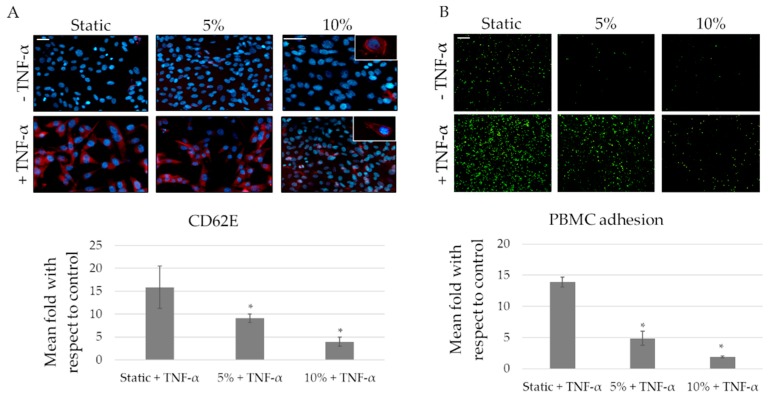
Strain affect inflammation mediated by endothelial cells (ECs). (**A**) Representative immunofluorescence staining for CD62E after mechanical (5 and 10%) and chemical (Tumor necrosis factor-alpha TNF-α 50ng/mL) stimulation. CD62E is observed in red while DAPI is used for nuclear staining. Quantification of positive cells expressing CD62E. Normalization of samples stimulated by TNF-α in relation to the respective control. Scale bar 30 µm * *p* ≤ 0.05; (**B**) Peripheral blood mononuclear cells (PBMCs) adhesion on ECs. PBMCs are observed in green. Normalization of samples stimulated by TNF-α in relation to the respective control (static, 5% and 10%). Scale bar 25 µm * *p* ≤ 0.05.

As leukocytes adhesion on endothelium is promoted by E-selectin (CD62E) expression on ECs membrane, we evaluated the effect of mechanical stress on CD62E as well (Figure 6A). As expected, E-selectin is upregulated in presence of TNF-α. Data show that the substrate deformation, both 5% and 10% stretching at 1Hz, significantly and proportionally counteracts TNF-α effects on CD62E expression (Figure 6A). In addition, data concerning monocytes adhesion nicely confirm these data. In fact, as shown in Figure 6B, the mechanical deformation, at both 5% and 10% at 1Hz, significantly inhibits the monocytes’ adhesion, also in presence of TNF-α.

### 2.6. Strain Affects MMP-9 Expression and Activity in ECs

**Figure 7:**
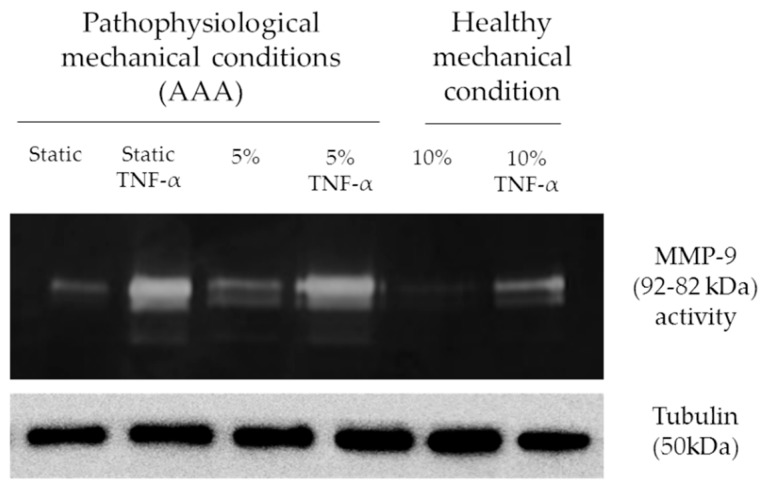
Strain affect MMP-9 expression and activity. Representative zymography assay to detect MMP-9 activity and expression after mechanical (5 and 10%) and chemical (TNF-α 50ng/mL) stimulation. Tubulin is used for loading control.

In static conditions, MMP-9 expression results strongly upregulated in presence of TNF-α. Applying a 5% substrate deformation, no significant inhibition of TNF-α effects is shown, resulting in an unmodified MMP-9 modulation (Figure 7), while no differences are found in absence of TNF-α with respect to static conditions. When a 10% substrate deformation is applied, MMP-9 expression results significantly downregulated in all conditions both in the absence and in the presence of TNF-α. 

## 3. Discussion

AAA is characterized by dramatic modifications of the medial layer, and it displays altered mechanical behavior, inflammatory response, and matrix remodeling in the aortic wall [29,30]. 

The calcification process has been acknowledged as a degenerative factor in inflammatory arterial diseases. Calcium deposits show a reverse correlation with aortic dilatation and inflammatory cell recruitment, as observed in our clinical data (results summarized in Table 2). However, the specific role of aortic dilatation in AAA progression is not completely elucidated. Taking together the results on AAA patients, it is evident that the decrease in dilatation is related to the presence of vascular calcifications in the medial layer. Moreover, the presence of calcification correlates with a decrease of MMP-9-promoted matrix remodeling. The severity of calcium deposition correlates also with IL-6-promoted inflammation, because in patients with high AAC index, most of cells, including also the inflammatory ones, are depleted and replaced by bone-like formation, resulting in a very low inflammatory infiltrate. Indeed, the higher levels of inflammation and matrix remodeling are found in patients with medium calcification index.

Inflammatory cell recruitment in AAA is sustained by the presence of inflammatory mediators and by the increased expression of adhesion molecules able to interact with circulating inflammatory cells [31]. Specifically, inflammatory cells infiltrate in the media and adventitia layers, inducing oxidative stress and over expression of cytokines/chemokines and MMPs. The findings obtained by proteomic analyses unveil also the involvement of oxidative stress in AAA patients, underlined by the over expression of the proteins implied in oxidation-reduction process and in cell response to oxidative stress. All these processes lead to elastic fiber breakdown, and depletion of vSMCs. As a result, the aortic wall is weakened because of decreased thickness and reduced mechanical function. The aortic wall cannot counteract the blood flow and pressure, and the aortic wall dilates to form AAA. Among the inflammatory mediators, TNF-α plays a pivotal role in the initiation and progression of vascular disorder by modulating the expression of molecules involved in vascular tone, inflammation, and remodeling, thus inducing endothelial dysfunction [32,33] and upregulating the adhesion molecules, such as CD62E. An inflammatory condition, which leads to endothelial dysfunction, contributes to the pathogenesis of vascular syndromes by predisposing vessels to plaque rupture and intravascular thrombosis [34]. Due to endothelial dysfunctions, thus, ECs also contribute to AAA progression [35]. 

In our study, we have tested two different percentages of substrate deformation on endothelial cell culture: 5% and 10% for three days at 1 Hz constant frequency, representing the resting heartbeat, while the substrate deformation of 10% is selected to mimic the dilation of the aortic wall under physiological conditions [36]. Our experimental settings are consistent with clinical findings on healthy individuals and on AAA patients with medium calcification index (Table 2).

The results suggest that 10% stimulation controls inflammation and ROS/RNS production compared to 5% dynamic and static samples; moreover, this experimental condition significantly contrasts TNF-α-mediated inflammatory effects. Thus, these observations highlight the importance of physiological vascular wall stretching as a powerful anti-inflammatory stimulus. Indeed, a downregulation of MMP-9, CD62E, and a decrease of peripheral blood mononuclear cells (PBMC) adhesion in 10% dynamic samples is observed. In TNF-α-stimulated static samples, MMP-9, CD62E, and PBMC adhesion increase, demonstrating that these markers are closely related to the chronic inflammation. Overall, our findings indicate the importance of physiological vascular wall stretching as a powerful anti-inflammatory stimulus able to inhibit the pathological progression of AAA.

## 4. Materials and Methods 

### 4.1. Patients and Healthy Donors’ Enrollment 

Abdominal aortic aneurysm tissues were provided by the Vascular Surgery Unit, Hospital Maggiore, Novara (Italy). AAA tissues were collected from 15 patients (100% male) subjected to open surgical repair (OSR); demographical and clinical data are reported in Table 3. All data and samples were collected from donors correctly informed for the use of excessive pathological material for diagnostic and research purpose according to the local institute’s regulation and policies based on Declaration of Helsinki (AVATAR, 1.0 – protocol 208/CE – CE 43/18, date of approval: 6 April 2018). Healthy aortic tissues were collected from autopsies (*n* = 5). Selection criteria included cause of death not associated with cardiovascular affections. Peripheral blood from healthy donors was collected from AVIS Novara in order to isolate PBMC’s (AVATAR, 1.0 – protocol 208/CE – CE 43/18, date of approval: 6 April 2018).

### 4.2. AAA Patients: Calcification Modeling and Wall Dilatation

Calcificated regions are captured after the CAT exam from 12 patients. The amount of calcification was evaluated through a score (AAC- from 0 to 8): AAC 0-1 are considered low calcification index, AAC 2-3-4 are medium index, and AAC 5-6 high index [13]. Geometries of AAAs are reconstructed, and images of the abdominal aorta are obtained from immediately distal to the renal arteries to immediately proximal to the iliac bifurcation during doppler ultrasound examination. Maximum AAA diameter, determined by B-MODE doppler ultrasound, is 72 mm. The values of aneurysm dilatation are obtained using the following formula

$$\frac{Aortic\;neck\;systole-aortic\;neck\;diastole}{aortic\;neck\;diastole}:\;\frac{aneurysm\;systole-aneurysm\;diastole}{aneurysm\;diastole}=0.1:x$$

Differences between systole and diastole of the aortic neck (healthy part) and the aneurysm are normalized with their respective diastole. The proportion is obtained comparing the differences with 10% that is the physiological measured dilatation of the healthy aorta.

### 4.3. Histological Analyses on Human Aortic Samples

AAA and control samples were fixed in neutral buffered formalin for 24 hours, and 5 µm-thick sections were cut from paraffin-embedded tissues. Briefly, rehydrated sections were treated with a 1% aqueous silver nitrate solution (Sigma Aldrich, Milan, Italy). Silver is deposited replacing the calcium reduced by the strong light, and thereby visualized as metallic silver. To counterstain the samples was also used a 5% sodium thiosulfate solution and 0.1% nuclear fast red solution (all from Sigma Aldrich, Milan, Italy). Calcium deposits and salts are detectable in black or brown-black, nuclei in red and cytoplasm in pink. Immunohistochemistry analysis using CD4, CD20, and CD68 for inflammatory cells (prediluted antibodies, Roche-Ventana, Tucson, AZ, USA) was performed. All images were acquired using Pannoramic MIDI 3DHISTECH and analyzed with Pannoramic Viewer software (3DHISTECH, Budapest, Hungary). For objective quantification of calcium content and inflammatory cells, ImageJ software was used.

### 4.4. Proteomic Analysis

Tissues obtained from AAA vessel and from healthy control vessel were lysed in radioimmunoprecipitation buffer (RIPA) (150 mM sodium chloride, 1% Triton X100, 0.5% sodium deoxycholate, 0.1% sodium dodecyl sulfate, SDS, 1 mM ethylenediaminetetraacetic acid, EDTA, 1 mM ethyleneglycol-bis(2-aminoethylether)-*N*,*N*,*N*′,*N*′-tetraacetic acid, EGTA, 50 mM Tris(hydroxymethyl)aminomethane TRIS pH = 7.4) supplemented with protease inhibitors (0.2 mM sodium othovanadate, 1 mM phenylmethyl sulfonyl fluoride and protease inhibitors cocktail, all from Sigma, Milan, Italy). Proteins concentration was determined using the bicinchoninic acid assay (Pierce, Rockford, IL, USA). Lysate proteins were digested using the following protocol: samples were subjected to denaturation with trifluoroethanol (TFE), to reduction with dithiothreitol (DTT) 200 mM, alkylation with iodoacetamide (IAM) 200 mM and the complete protein trypsin digestion with 2 μg of Trypsin/Lys-C (Promega, Madison, WI, USA). The peptide digests were desalted on the Discovery^®^ DSC-18 solid phase extraction (SPE) 96-well Plate (25 mg/well) (Sigma-Aldrich Inc., St. Louis, MO, USA). Peptides were dried by speed vacuum until the analysis. 

Liquid chromatography– tandem mass spectrometry (LC–MS/MS) analyses were performed on digests using a micro-LC Eksigent Technologies (Dublin, OH, USA) system with a stationary phase of a Halo Fused C18 column (0.5 × 100 mm, 2.7 μm; Eksigent Technologies, Dublin, OH, USA). The injection volume was 4.0 μL and the oven temperature was set at 40 °C. The mobile phase was a mixture of 0.1% (*v*/*v*) formic acid in water (A) and 0.1% (*v*/*v*) formic acid in acetonitrile (B), eluting at a flow-rate of 15.0 μL min^−1^ at an increasing concentration of solvent B from 2% to 40% in 30 min. LC system was interfaced with a 5600 + TripleTOF (Time of flight) system (AB Sciex, Concord, ON, Canada) equipped with a DuoSpray Ion Source and Calibrant Delivery System (CDS). The relative abundance of proteins was obtained using the label-free quantification. Samples were subjected to data-dependent acquisition (DDA): the mass spectrometer analysis was performed using a mass range of 100–1500 Da TOF scan with an accumulation time of 0.25 s), followed by a MS/MS product ion scan from 200 to 1250 Da (accumulation time of 5.0 ms) with the abundance threshold set at 30 cps (35 candidate ions can be monitored during every cycle). The samples were, then, subjected to cyclic data independent analysis (DIA) of the mass spectra, using a 25-Da window: the mass spectrometer was operated such that a 50-ms survey scan (TOF-MS) was performed and subsequent MS/MS experiments were performed on all precursors [20,21]. The MS data were acquired with Analyst TF 1.7 (AB SCIEX, Concord, ON, Canada). Three instrumental replicates for each sample were subjected to the DIA analysis. 

Protein identification was performed using Mascot v. 2.4 (Matrix Science Inc., Boston, MA, USA), the digestion enzyme selected was trypsin, with two missed cleavages and a search tolerance of 50 ppm was specified for the peptide mass tolerance, and 0.1 Da for the MS/MS tolerance. The charges of the peptides to search for were set to 2+, 3+, and 4+, and the search was set on monoisotopic mass. The instrument was set to electrospray ionization – quadrupole- time of flight (ESI-QUAD-TOF) and the following modifications were specified for the search: carbamidomethyl cysteines as fixed modification and oxidized methionine as variable modification. The UniProt Swiss-Prot reviewed database containing human proteins (version 2015.07.07, containing 42131 sequence entries) was used and a target-decoy database search was performed. False Discovery Rate was fixed at 1%. The label-free quantification was carried out with PeakView 2.0 and MarkerView 1.2. (ABSCIEX, Concord, ON, Canada). The upregulated proteins were selected using *p* value < 0.05 and fold change >1.5. The upregulated proteins were analyzed by using STRING software (http://string-db.org), which is a database of known and predicted protein–protein interactions.

### 4.5. Dynamic Cell Culture

Human endothelial cells, EA.hy926 (ATCC^®^ CRL-2922™, Manassas, VA, USA) were cultured in high-glucose Dulbecco’s modified Eagle’s medium (DMEM) enriched with 10% fetal bovine serum and penicillin (100 U/mL), streptomycin (100 µg/mL), and 2 mM glutamine mixture (all from Euroclone, Milan, Italy) at 37 °C in humid 5% CO2 atmosphere. Rectangular silicone pieces (3 cm x 1.5 cm) were cut and sterilized together with the culture chambers of the TC-3 bioreactor (Ebers Medical, Zaragoza, Spain). To facilitate cell adhesion to the silicone substrate (both static controls and dynamic samples) a type I collagen (50 μg/mL) coating was used. After one hour, the coating has been rinsed with sterile water to remove the exceeding collagen. Finally, Ea.hy926 (2 × 10^4^ /cm^2^) were seeded, and after 24 h (required for an optimal adhesion to the substrate), mechanical stimulation has been applied to the cell culture. A stretching of 5 and 10% was maintained for 72 h, 50 ng/mL of TNF-α (Sigma Aldrich, Milan, Italy) was added when required. Silicone controls were maintained under static conditions.

### 4.6. Phalloidin Staining

Cells were fixed in formalin 4% and incubated with phalloidin-tetramethylrhodamine conjugated (TRITC) (Sigma Aldrich, Milan, Italy) for 45’ at 37 °C. 4′,6-Diamidine-2′-phenylindole dihydrochloride (DAPI Sigma Aldrich, Milan, Italy) was used for nuclear staining. Samples were observed at fluorescent microscope (DM2500 Leica, Wetzlar, Germany).

### 4.7. Cellular ROS/Superoxide Detection Assay Kit

Oxidative stress production was investigated through Cellular ROS/Superoxide detection assay kit (ab139476, Abcam, Cambridge, UK) following the manufacturer’s protocol. Briefly, detached cells (static controls, 5%, and 10% of mechanical stimulation) were stained with oxidative stress reagent orange and green, then flow cytometry analyses were performed (ATTUNE NxT Cytometer, Thermo Fisher, Waltham, MA, USA) and analyzed by ATTUNE NxT flow cytometer software. Pyocyanin treated cells (400 µM) were used as positive control. 

### 4.8. Immunofluorescence 

Immunofluorescence analyses were carried out on mechanically stimulated silicone cells and on static controls. 1 × 10^4^ EA.hy926/cm^2^ were seeded and maintained in culture according to experimental protocols. After three days of stimulation, samples were fixed for one hour with 4% formalin. Samples were blocked for one hour with a 5% goat solution (Euroclone, Milan, Italy) and 0.3% TRITON X-100 in phosphate buffer saline (PBS; Sigma Aldrich, Milan, Italy) and, then, incubated with the primary antibody (1:50 anti-E-selectin, Santa Cruz Biotechnology, Dallas, TX, USA) for one hour at room temperature. E-selectin is detected by a secondary antibody TRITC-conjugated (Perkin-Elmer, Milan, Italy) and observed at fluorescent microscope (DM2500 Leica, Germany). The images were acquired using Leica acquisition software (Leica, Wetzlar, Germany). Data are expressed as TNF-alpha-treated versus the respective untreated samples ratio. 

### 4.9. Leukocyte-Endothelium Adhesion Assay

PBMCs were isolated with Histopaque^®^-1077 (Sigma Aldrich, Milan, Italy) from peripheral blood obtained by healthy donors. PBMCs adhesion assay was performed using Cell Biolabs’ CytoSelectTM Leukocyte-endothelium Adhesion Assay (Cell Biolabs Inc., San Diego, CA, USA). After mechanical stimulations, PBMCs were labeled by the LeukoTrackerTM solution. Labeled PBMCs were then incubated with static and dynamic cells in presence or not of TNF-α (50 ng/mL). After one hour of incubation, nonadherent cells were removed by gently rinsing with PBS. Adherent cells were counted in three separate fields using an inverted fluorescence microscope (DM2500 Leica, Wetzlar, Germany). Data are expressed as TNF-alpha-treated versus the respective untreated samples ratio. 

### 4.10. Western Blot 

Culture cells and tissues were lysed in RIPA buffer supplemented with protease inhibitors. Proteins concentration was determined using the bicinchoninic acid assay (Pierce, Waltham, IL, USA). 50 μg total proteins in sample buffer (62.5 mM Tris-HCl, pH 6.8, 20% glycerol, 5% β-mercaptoethanol, 0.5% bromophenol blue; all from Sigma Aldrich, Milan, Italy) were resolved to SDS-polyacrylamide gel electrophoresis (SDS-PAGE) and transferred to a nitrocellulose membrane (Amersham Biosciences, Little Chalfont, UK). Membranes were incubated overnight with IL-6, OPN (Abcam, Cambridge, UK), MMP-9, Tubulin (Millipore, Milan, Italy) antibodies at 4 °C. Proteins were revelated with secondary antibody-peroxidase conjugates (Perkin-Elmer, Milan, Italy). Protein bands were visualized using enhanced chemiluminescence (ECL, Perkin-Elmer, Western lightning PLUS-ECL, Milan, Italy) detection reagents in a chemosensitive visualizer (VersaDoc, BioRad, Milan, Italy). In order to check the loaded proteins concentration, red ponceau (Sigma Aldrich, Milan, Italy) staining was considered. 

### 4.11. Zymography Assay 

Non-reduced protein samples were resolved by SDS-PAGE gels containing type A gelatin from porcine skin (0.2%, Sigma Aldrich, Milan, Italy). Briefly, after electrophoresis, gels were incubated with a solution of 2.5% TRITON X-100 for 3 h at room temperature, and then incubated in a solution of calcium chloride (CaCl_2_, 1 mM) and sodium chloride (NaCl, 15 mM), pH 7.4 (all from Sigma Aldrich, Milan, Italy) overnight at 37 °C. Subsequently, gels were fixed and then stained with Coomassie Blue (Sigma Aldrich, Milan, Italy). For objective quantification ImageJ software was used.

### 4.12. Statistical Analyses

All experiments were performed in triplicate. All data are expressed as mean values ± standard deviation. Using Student’s *t*-test, the *p*-value is calculated and the differences between variables with a value of *p* < 0.05 are considered statistically significant.

## 5. Conclusions

In conclusion, we found a negative correlation between calcium deposits and wall dilatation in presence of AAA. A decreased wall stretching, due to the presence of calcification, affects MMP-9-mediated matrix degradation and IL-6-mediated inflammation. As expected, *in vitro* model on endothelial cell line shows that substrate deformation significantly regulates the inflammatory response and matrix remodeling. 

## Figures and Tables

**Table 1:** Proteomic analyses results. Upregulated proteins in AAA vessel tissue after comparison to healthy tissues by proteomic analysis. The upregulated proteins were selected using *p* value < 0.05.

Protein	Accession Name	Fold Change (*p*-Value < 0.05) AAA/Healthy	Biological Function
Protein disulfide-isomerase	PDIA1_HUMAN	2.50	oxidation-reduction process
Isoform H14 of Myeloperoxidase	PERM_HUMAN	3.04	oxidation-reduction process
Superoxide dismutase [Mn], mitochondrial	SODM_HUMAN	6.95	cell response to oxidative stress, oxidation-reduction process, removal of superoxide radicals
Ceruloplasmin	CERU_HUMAN	12.83	oxidation-reduction process
Ferritin heavy chain	FRIH_HUMAN	21.83	oxidation-reduction process
Ferritin light chain	FRIL_HUMAN	26.43	oxidation-reduction process
Catalase	CATA_HUMAN	2.51	cell response to oxidative stress

**Table 2:** Summary of obtained data. It shows the results obtained on AAA tissue in terms of calcium accumulation, dilatation, inflammation value, and matrix remodeling.

	Low AAC Index	Medium AAC Index	High AAC Index
calcium deposits	+	++	+++
dilatation (%)	5 < X < 10	≃ 5	X < 5
inflammation	moderate	high	Low
ECM remodeling	moderate	high	Moderate

**Table 3:** Demographical and clinical feature of AAA patients. It shows demographical data (age, sex) and cardiovascular risk (DAAA aneurysm diameters, hypercholesterolemia, smoking, hypertension, and ischemic cardiomyopathy). Age and DAAA are represented as mean ± standard deviation. Patient data are divided by the grade of aortic calcification index (AAC).

Patients	Age Mean ± SD	Gender	DAAA Mean ± SD	Hypercholesterolemia	Smoking	Hypertension	Ischemic Cardiomyopathy
**Low AAC**	72 ± 4	Male 100%	5.6 ± 1.4	100%	33%	100%	66%
**Medium AAC**	75 ± 6	Male 100%	5.4 ± 1.3	50%	50%	88%	50%
**High AAC**	71 ±12	Male 100%	5.3 ± 0.6	100%	33%	100%	66%

## Abbreviations

AAA	Abdominal aortic aneurysm
ECs	Endothelial cells
TNF-α	Tumor necrosis factor alpha
MMP-9	Matrix metalloproteinase -9
ECM	Extracellular matrix
PTM	Transmural pressure
vSMCs	Vascular smooth muscle cells
IL-1	Interleukin-1
IL-6	Interleukin-6
IL-8	Interleukin-8
MCP-1	Monocyte chemoattractant protein 1
RANTES	Regulated on activation, normal T cell expressed and secreted
CAMs	Cell adhesion molecules
ROS	Reactive oxygen species
NADPH	Nicotinamide adenine dinucleotide phosphate
NOX	Nicotinamide adenine dinucleotide phosphate oxidase
XO	Xanthine oxidase
SOD	Superoxide dismutase
TRX	Thioredoxin
MMPs	Matrix metalloproteinases
NF-kB	Nuclear factor kappa-light-chain-enhancer of activated B cells
AP-1	Activator protein 1
CAT	Computed axial tomography
CDs	Clusters of differentiation
AAC	Aortic aneurysm calcification
OPN	Osteopontin
LC-MS	Liquid chromatography-mass spectrometry
CATA	Catalase
SODM	Superoxide dismutase (Mn), mitochondrial
PDIA1	Protein disulfide-isomerase
PERM	Isoform H14 of myeloperoxidase
CERU	Ceruloplasmin
FRIH	Ferritin heavy chain
FRIL	Ferritin light chain
RNS	Reactive nitrogen species
FACS	Fluorescence-activated cell sorting
CD62E	E-selectin
PBMCs	Peripheral blood mononuclear cells
OSR	Open surgical repair
DAAA	Diameter of abdominal aortic aneurysm
RIPA	Radioimmunoprecipitation buffer
EDTA	Ethylenediaminetetraacetic acid
EGTA	Ethyleneglycol-bis(2-aminoethylether)-*N*,*N*,*N*′,*N*′-tetraacetic acid
TFE	Trifluoroethanol
DTT	Dithiothreitol
IAM	Iodoacetamide
SPE	Solid phase extraction
LC-MS/MS	Liquid chromatography-tandem mass spectrometry
TOF	Time of flight
CDS	Calibrant delivery system
DDA	Data-dependent acquisition
DIA	Data independent analysis
ESi-QUAD-TOF	Electrospray ionization-quadrupole-time of flight
DMEM	Dulbecco’s modified Eagle’s medium
TRITC	Tetramethylrhodamine
DAPI	4′,6-Diamidine-2′-phenylindole dihydrochloride
PBS	Phosphate buffered saline
SDS-PAGE	Sodium dodecyl sulfate polyacrylamide gel electrophoresis
